# Time trend prediction and spatial–temporal analysis of multidrug-resistant tuberculosis in Guizhou Province, China, during 2014–2020

**DOI:** 10.1186/s12879-022-07499-9

**Published:** 2022-06-07

**Authors:** Wang Yun, Chen Huijuan, Liao Long, Lu Xiaolong, Zhang Aihua

**Affiliations:** 1 Key Laboratory of Environmental Pollution Monitoring and Disease Control, Ministry of Education, School of Public Health, Guizhou Medical University, Guiyang, Guizhou China; 2 Department of Tuberculosis Prevention and Control, Guizhou Center for Disease Prevention and Control, Guiyang, Guizhou China; 3 School of Medicine and Health Management, Guizhou Medical University, Guiyang, Guizhou China

**Keywords:** MDR-TB, Prediction, SARIMA model, Spatial−temporal analysis

## Abstract

**Background:**

Guizhou is located in the southwest of China with high multidrug-resistant tuberculosis (MDR-TB) epidemic. To fight this disease, Guizhou provincial authorities have made efforts to establish MDR-TB service system and perform the strategies for active case finding since 2014. The expanded case finding starting from 2019 and COVID-19 pandemic may affect the cases distribution. Thus, this study aims to analyze MDR-TB epidemic status from 2014 to 2020 for the first time in Guizhou in order to guide control strategies.

**Methods:**

Data of notified MDR-TB cases were extracted from the National TB Surveillance System correspond to population information for each county of Guizhou from 2014 to 2020. The percentage change was calculated to quantify the change of cases from 2014 to 2020. Time trend and seasonality of case series were analyzed by a seasonal autoregressive integrated moving average (SARIMA) model. Spatial–temporal distribution at county-level was explored by spatial autocorrelation analysis and spatial–temporal scan statistic.

**Results:**

Guizhou has 9 prefectures and 88 counties. In this study, 1,666 notified MDR-TB cases were included from 2014–2020. The number of cases increased yearly. Between 2014 and 2019, the percentage increase ranged from 6.7 to 21.0%. From 2019 to 2020, the percentage increase was 62.1%. The seasonal trend illustrated that most cases were observed during the autumn with the trough in February. Only in 2020, a peak admission was observed in June. This may be caused by COVID-19 pandemic restrictions being lifted until May 2020. The spatial–temporal heterogeneity revealed that over the years, most MDR-TB cases stably aggregated over four prefectures in the northwest, covering Bijie, Guiyang, Liupanshui and Zunyi. Three prefectures (Anshun, Tongren and Qiandongnan) only exhibited case clusters in 2020.

**Conclusion:**

This study identified the upward trend with seasonality and spatial−temporal clusters of MDR-TB cases in Guizhou from 2014 to 2020. The fast rising of cases and different distribution from the past in 2020 were affected by the expanded case finding from 2019 and COVID-19. The results suggest that control efforts should target at high-risk periods and areas by prioritizing resources allocation to increase cases detection capacity and better access to treatment.

## Introduction

Multidrug-resistant tuberculosis (MDR-TB) is a specific type of pulmonary tuberculosis (PTB) and a chronic infectious respiratory disease. MDR-TB is caused by *Mycobacterium tuberculosis* (MTB) which is resistant to at least two anti-TB drugs: isoniazid and rifampin [1]. It was estimated that there were 362,700 MDR-TB cases worldwide in 2019 and China shares the second largest global MDR-TB caseloads after India [1]. The number of MDR-TB patients enrolled and started treatment in China increased from 5691 in 2015 [2] to 5943 in 2017 [1]. The improvement of cases notifications may due to China expanded MDR-TB care after the completion of the Global Fund programme in 2014 [3]. While, the growing epidemic of MDR -TB also pose a serious threat to TB control efforts and achieving post-2015 global TB targets in China [4].

Guizhou is located in the southwest of China with 9 prefectures and 88 counties. It is a low-income province in China with the second largest rural population and the third highest TB incidence [5, 6]. The total multi-drug resistance rate in Guizhou (14.3%) was higher than the national average level (8.32%) reported from the national TB epidemiological sampling survey in 2010 [7]. MDR-TB remains a big challenge to public health in Guizhou. Therefore, followed the National TB Control Program, Guizhou provincial authorities have made efforts to establish gradually MDR-TB health service system and perform the strategies for active case finding among high risk groups for drug-resistant TB [8] since 2014. From 2014 to 2020, 33,396 high-risk people were screened by drug susceptibility testing (DST). Of all, 25,130 people (75.2%) were screened in 2019 and 2020 [9]. In pace with these actions, MDR-TB cases detection may be improved yearly. While, COVID-19 pandemic may affect TB services [10, 11] and create a different distribution of cases in 2020. Hence, it is necessary to identify the MDR-TB epidemic status in recent years in order to provide some information for further MDR-TB control in Guizhou.

Time series and spatial–temporal analysis are powerful in characterize infectious disease epidemics. They have been adopted to detect the regular distribution of diseases or abnormal fluctuation due to unpredictable factors. The seasonal autoregressive integrated moving average model (SARIMA) is one of the most representative methods and widely applied in time series analysis of PTB [12–17] and other infectious disease [18–20]. Meanwhile, spatial autocorrelation analysis and spatial–temporal scan statistic are popularly used to explored the spatial–temporal heterogeneity of PTB in recent years [14, 21–24]. However, only limited studies explored the current status of MDR-TB [25, 26]. Hence, this study aims to clarify the epidemic characteristics of MDR-TB from 2014 to 2020 by above methods for the first time in Guizhou province.

## Methods

### Date source

The total number of high-risk group screened by DST in 2020 and the MDR-TB cases data were extracted from National TB Surveillance System. The database used in this study was obtained from the Institute of Tuberculosis Control and Prevention of Guizhou Provincial Center for Disease Control and Prevention. The epidemiological information of each case was collected, including: age, gender, ethnic, occupation, anti-TB drug history, current home address, date of diagnosis, date of registration and date of start treatment. Population data for each county at year-end were collected from yearbook of Guizhou Provincial Bureau of Statistics. Fundamental geographic data were downloaded from the National Geomatics Centre of China to make the county-level polygon map of Guizhou, and all cases were geocoded and matched to the map.

### Data analysis

#### Descriptive analysis for general characteristics

The percentage change was calculated to quantify the change of annual MDR-TB cases from 2014 to 2020. Percentage change = [ (final value – starting value) / |starting value|] × 100. Epidemic characteristics of MDR-TB cases were analyzed at provincial level from 2014 to 2020 by descriptive statistics, including the distribution of gender, age, ethnic group, occupation, anti-TB drug history. The number of MDR-TB cases was descriptively analyzed at prefecture level in each year. These descriptive analyses were performed by R (version 4.1.0, Vienna, Austria). The geographical distribution of total MDR-TB cases was descriptively analyzed at county level by ArcGIS v.10 software (ESRI Inc., Redlands, CA, USA).

#### Time series analysis

Time series was broken down first by decomposition approach into its trend, seasonality and randomness [17, 27]. The seasonal index (SI) was calculated to verify seasonality [28, 29]. Then, the SARIMA model was used to perform time-series analysis. The SARIMA model is generally formed as: SARIMA (p, d, q) (P, D, Q)s, where p, d and q are, respectively, the auto-regressive (AR) order, the number of the differences necessary to achieve stationarity and moving average (MA) order; P, D and Q are the corresponding seasonal orders; S represents the specific value of seasonal period. The period of TB in China is 12 months (s = 12) [30].

The data of MDR-TB cases from January 2014 to December 2019 was used as a training dataset, and that data from January to December of 2020 was used as the forecasting dataset. Three steps are used to establish the SARIMA mode: (1) checking stationarity of the training dataset by augmented Dickey−Fuller method; (2) constructing models based on the autocorrelation function (ACF) and partial autocorrelation function (PACF) of the model residuals, then testing residuals of ACF and PACF by Ljung-Box Q test, and selecting the optimal model with lowest Akaike information criterion (AIC) and Bayesian information criterion (BIC) from some candidate models; (3) measuring the accuracy of the forecasts by comparing the predicted values with the observed values, then applying the model further to forecast the cases in 2021 and 2022, respectively.

#### Spatial–temporal analysis

Spatial–temporal analysis was explored by spatial autocorrelation analysis and spatial–temporal scan statistic at county level.

First, spatial autocorrelation analysis mainly includes global autocorrelation analysis [31, 32] and local autocorrelation analysis [33, 34], and performed by ArcGIS. Global Moran’s I value was used to identify spatial autocorrelation and detect the spatial distribution pattern of disease. Moran’s I ranges from − 1 to 1, when the value is positive and close to 1 indicates that the correlation is positive and more obvious of clustering. While, when the value is negative and close to − 1 indicates that the correlation is negative and more discrete. When the value is 0 means a random distribution of disease. The significance of Moran’s I was evaluated by Z-score and P-value. Based on Moran’s I and its significance, four types of clusters can be detected by local autocorrelation analysis, including the high–high (HH), low–low (LL), high–low (HL) and low–high (LH) clustering patterns, respectively. HH clusters indicate areas with a high MDR-TB notification surrounded by areas with similarly high MDR-TB notification, while LL clusters indicate the opposite of HH clusters. HL outliers specifies areas with high MDR-TB notification surrounded by areas with low notification, while LH outliers designate the opposite of HL clusters. The significance level was 0.05, and the number of permutations was 999.

Second, spatial–temporal scan statistic was performed by SaTScan™ User Guide version 10.0 (Martin Kulldorff, July 2021) [35], and was used to clarify the clusters of disease across different regions geographically and in different year. This method is defined by a moving cylindrical window that includes the circular base and the cylinder height. The circular base indicates a geographical area and the height reflects the time period. The cylindrical window was moved on the map to detect possible spatial–temporal clusters based on the Poisson probability model. The number of expected cases was calculated based on the number of actual observed cases and the number of population. The specific clusters were identified by calculating the logarithmic likelihood ratio (LLR) to compare the observed and expected numbers of cases. Monte Carlo simulation test was used to evaluate whether the difference is statistically significant. The window with the maximum LLR value was the most likely cluster and other statistically significant windows were secondary clusters. In this analysis, the largest spatial size of the clusters was set to 50% of the total population at risk and the largest temporal size was 50% of the total study period. Finally, the scanning results was visualized by ArcGIS software.

### Ethical review

In this study, MDR-TB data were collected from routine TB surveillance system. The written permission to use these data and approval of the study were obtained from the Institute of Tuberculosis Control and Prevention of Guizhou Provincial Center for Disease Control and Prevention.

## Results

### General characteristics of MDR-TB patients

**Fig. 1:**
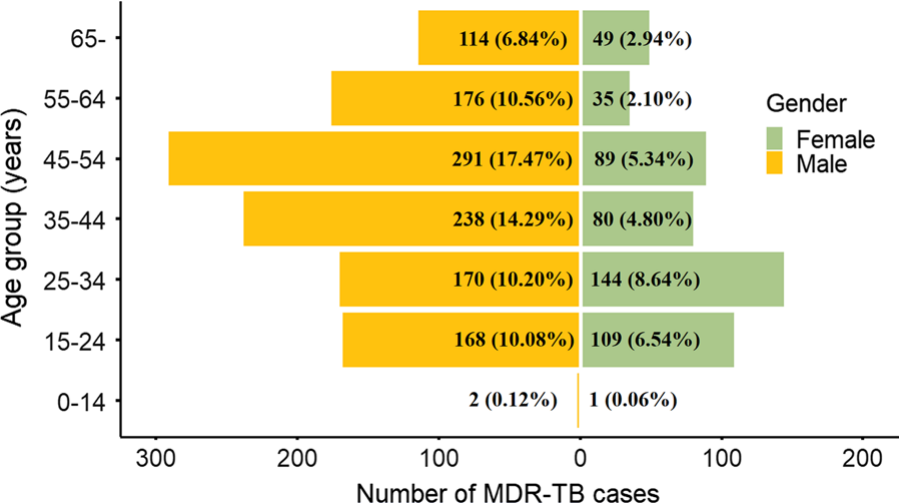
MDR-TB cases reported disaggregated by age and gender from 2014 to 2020

A total of 1,666 cases of MDR-TB were reported in Guizhou Province from 2014 to 2020. The number of cases increased yearly. Between 2014 and 2019, the percentage increase in the number of cases ranged from 6.7 to 21.0%. From 2019 to 2020, the percentage increase was 62.1%. There were 69.6% male and 30.4% female with a 2.3 sex ratio. (Table 1) MDR-TB affected all age groups and each group with more male than female cases (Fig. 1). The highest burden was among young to middle-age adult men who accounted for 62.6% (1043) of all cases, compared with 27.4% (457) of adult women and 0.2% (3) in children. In terms of ethnic and occupational distribution, 82.5% cases were Han, and the cases were mainly farmers (43.6%) followed by migrant workers (32.4%) and others (16.5%). Of all cases, only 4.8% cases never used any anti-TB drugs before this diagnosis of MDR-TB, while 87.8% experienced first-line and 7.4% experienced first- and second-line anti-TB drugs treatment.

**Table 1:** Demographic characteristics of MDR-TB cases in Guizhou Province, 2014–2020 (n, %)

Variables	2014	2015	2016	2017	2018	2019	2020	Total
Total	145	165	186	225	240	269	436	1666
Percentage change (%)	–	13.8	12.7	21.0	6.7	12.1	62.1	–
*Gender*								
Male	101 (69.7)	125 (75.8)	130 (69.9)	142 (63.1)	173 (72.1)	177 (65.8)	311 (71.3)	1159 (69.6)
Female	44 (30.3)	40 (24.2)	56 (30.1)	83 (36.9)	67 (27.9)	92 (34.2)	125 (28.7)	507 (30.4)
Sex ratio	2.3	3.1	2.3	1.7	2.6	1.9	2.5	2.3
*Age*								
< 25	24 (16.6)	28 (17.0)	35 (18.8)	50 (22.2)	43 (17.9)	32 (11.9)	68 (15.6)	280 (16.8)
25–65	114 (78.6)	125 (75.8)	141 (75.8)	159 (70.7)	168 (70.0)	213 (79.2)	303 (69.5)	1223 (73.4)
≥ 65	7 (4.8)	12 (7.3)	10 (5.4)	16 (7.1)	29 (12.1)	24 (8.9)	65 (14.9)	163 (9.8)
*Ethnic group*								
Han	127 (87.6)	141 (85.5)	167 (89.8)	200 (88.9)	185 (77.1)	212 (78.8)	342 (78.4)	1374 (82.5)
Others	18 (12.4)	24 (14.5)	19 (10.2)	25 (11.1)	55 (22.9)	57 (21.2)	94 (21.6)	292 (17.5)
*Occupation*								
Student	6 (4.1)	1 (0.6)	1 (0.5)	10 (4.4)	15 (6.3)	4 (1.5)	20 (4.6)	57 (3.4)
Government worker	16 (11.0)	4 (2.4)	4 (2.2)	2 (0.9)	15 (6.3)	15 (5.6)	12 (2.8)	68 (4.1)
Farmer	68 (46.9)	92 (55.8)	111 (59.7)	104 (46.2)	104 (43.3)	86 (32.0)	161 (36.9)	726 (43.6)
Migrant worker	13 (9.0)	8 (4.8)	13 (7.0)	51 (22.7)	72 (30.0)	154 (57.2)	229 (52.5)	540 (32.4)
Others (unemployed)	42 (29.0)	60 (36.4)	57 (30.6)	58 (25.8)	34 (14.2)	10 (3.7)	14 (3.2)	275 (16.5)
*Anti-TB drug history*								
Never use	0 (0.0)	0 (0.0)	0 (0.0)	16 (7.1)	21 (8.8)	15 (5.6)	28 (6.4)	80 (4.8)
Only 1st-line drugs	114 (78.6)	162 (98.2)	174 (93.5)	193 (85.8)	187 (77.9)	235 (87.4)	397 (91.1)	1462 (87.8)
1st- and 2nd-line drugs	31 (21.4)	3 (1.8)	12 (6.5)	16 (7.1)	32 (13.3)	19 (7.1)	11 (2.5)	124 (7.4)

*MDR-TB* multidrug-resistant tuberculosis, *anti-TB* anti-tuberculosis

### Time-series analysis

**Fig. 2:**
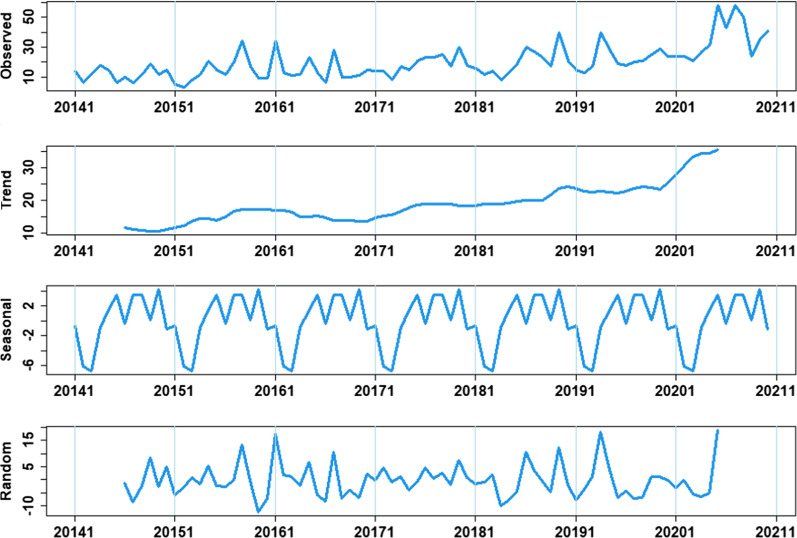
Time series decomposition of MDR-TB cases from January 2014 to December 2020

**Fig. 3:**
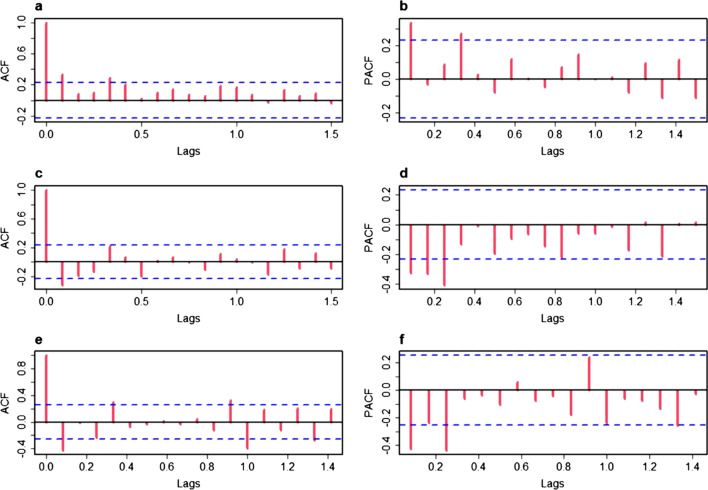
The ACF and PACF graphs for estimating the parameter. **a** The ACF graph of the raw data (d = 0 and D = 0); **b** the PACF graph of the raw data (d = 0 and D = 0); **c** the ACF graph of one-order trend difference data (d = 1 and D = 0); **d** the PACF graph of one-order trend difference data (d = 1 and D = 0); **e** the ACF graph of one-order seasonal difference data (d = 1 and D = 1); **f** the PACF graph of one-order seasonal difference data (d = 1 and D = 1)

**Fig. 4:**
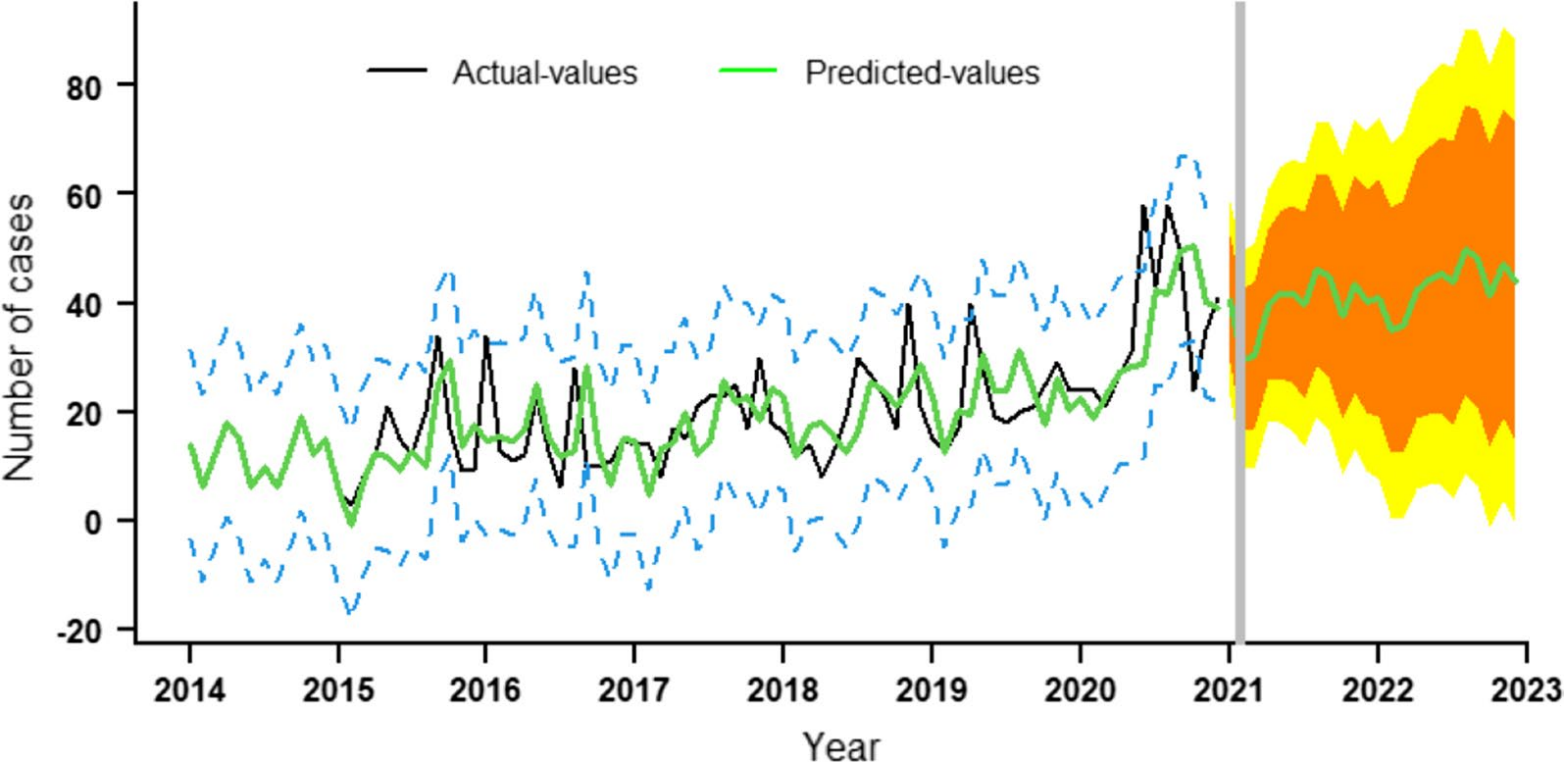
Comparison of actual and predicted cases of MDR-TB in Guizhou China. the black and green lines represent the observed values and predicted values, respectively; blue line represents 95% confidence intervals; after grey vertical line, the orange and yellow part represent 80% and 95% confidence intervals, respectively

Time series presented obvious seasonality, periodicity and randomness after being decomposed (Fig. 2). The results of SI further confirmed that there was a obvious seasonality among MDR-TB cases in Guizhou Province, with the trough usually occurring in February and the trend of gradually increasing from March to September, then declining from October (Table 2). So, the SARIMA model was used to analyse the time-series of MDR-TB in Guizhou from 2014 to 2020. First, using the raw training data from January 2014 to December 2019, trend difference (d = 1) and seasonal difference (D = 1) were calculated (Fig. 3). The augmented Dickey–Fuller method indicated that the original series was not stationary (t = − 3.1091, p = 0.121) but the series with both transformations (d = 1 and D = 1) was stationary (t = − 5.798, p < 0.01). Second, the ACF and PACF plots were used to estimate the ranges of four parameters (p, q, P, Q). Then, six candidate SARIMA models were used to be compare to find the optimal model. On the basis of the results of the goodness-of-fittest statistics, SARIMA(3,1,0)(0,1,1)_12_ was the best fitted model with lowest AIC and BIC values, and also passed the Ljung–Box Q Test (p = 0.444). All the parameter estimates were significant (Table 3). Finally, the model SARIMA (3,1,0)(0,1,1)_12_ forecasting effect was tested by comparing the predicted values with the observed values from January to December 2020 (Table 4). Except for actual values in June and August, the remaining actual values are among 95% CI of predicted values. The predicted trend was basically consistent with the actual trend from 2014 to 2019 demonstrating that the model prediction results were reliable (Fig. 4). Based on this model, the number of MDR-TB reported cases in Guizhou in 2021 and 2022 was predicted to be 475 (175 ~ 776) and 518 (53 ~ 987), respectively.

**Table 2:** The SI of MDR-TB cases distribution in each month from 2014 to 2020 in Guizhou Province

Month	Average number of cases per month	SI
January	17	0.87
February	12	0.61
March	13	0.65
April	19	0.96
May	21	1.04
June	22	1.08
July	20	1.01
August	26	1.30
September	25	1.25
October	18	0.92
November	24	1.19
December	20	1.02

*SI* seasonal index, *MDR-TB* multidrug-resistant tuberculosis

**Table 3:** Comparison of candidate SARIMA models

Model	Estimate	t	p	Ljung-Box Q Test		AIC	BIC	RMSE	MAPE
				Statistics	p-Value				
SARIMA(3,1,0)(0,1,1)_12_				0.585	0.444	435.490	445.873	6.684	29.372
AR1	− 0.596	5.119	< 0.001						
AR2	− 0.525	4.235	< 0.001						
AR3	− 0.427	3.683	< 0.001						
SMA1	− 1.000	2.607	0.006						
SARIMA(3,1,0)(1,1,1)_12_				0.575	0.448	437.480	449.948	6.696	29.405
AR1	− 0.597	5.114	< 0.001						
AR2	− 0.523	4.046	< 0.001						
AR3	− 0.427	3.675	< 0.001						
SAR1	0.010	0.055	0.478						
SMA1	− 1.000	2.721	0.004						
SARIMA(3,1,0)(0,1,2)_12_				0.576	0.448	437.480	449.948	6.694	29.401
AR1	− 0.597	5.114	< 0.001						
AR2	− 0.524	4.059	< 0.001						
AR3	− 0.427	3.676	< 0.001						
SMA1	− 0.991	2.431	0.009						
SMA2	− 0.009	0.053	0.479						
SARIMA(3,1,0)(2,1,0)_12_									
AR1	− 0.568	4.873	< 0.001	0.669	0.413	440.750	453.213	7.825	33.859
AR2	− 0.524	4.229	< 0.001						
AR3	− 0.441	3.840	< 0.001						
SAR1	− 0.668	4.233	< 0.001						
SAR2	− 0.295	1.909	0.030						
SARIMA(3,1,0)(2,1,2)_12_									
AR1	− 0.600	5.174	< 0.001	0.661	0.416	441.130	457.748	6.471	28.293
AR2	− 0.531	4.105	< 0.001						
AR3	− 0.438	3.760	< 0.001						
SAR1	− 0.967	5.296	< 0.001						
SAR2	− 0.064	0.304	0.381						
SMA1	0.000	0.000	0.500						
SMA2	− 1.000	1.910	0.030						
SARIMA(3,1,0)(2,1,3)_12_				0.857	0.355	441.500	460.203	5.978	26.217
AR1	− 0.596	5.049	< 0.001						
AR2	− 0.563	4.497	< 0.001						
AR3	− 0.461	4.003	< 0.001						
SAR1	− 1.734	12.962	< 0.001						
SAR2	− 0.946	5.593	< 0.001						
SMA1	0.983	1.107	0.136						
SMA2	− 0.643	0.428	0.335						
SMA3	− 0.881	1.026	0.154						

*AIC* Akaike information criterion, *BIC* Bayesian information criterion, *RMSE* root mean square error, *MAPE* mean absolute percent error

**Table 4:** Comparison of actual values and predicted values from January to December 2020

Month	Actual value	Predicted value	95%CI	
			LCL	UCL
January	24	22	6	37
February	24	18	1	35
March	21	20	2	37
April	27	26	8	44
May	31	26	6	46
June	58	23	2	44
July	43	24	3	46
August	58	28	6	51
September	50	28	5	51
October	24	25	1	49
November	35	30	5	54
December	41	25	1	50

*LCL* lower confidence limit, *UCL* upper confidence limit

### Spatial–temporal analysis

#### The geographical distribution of MDR-TB cases

**Fig. 5:**
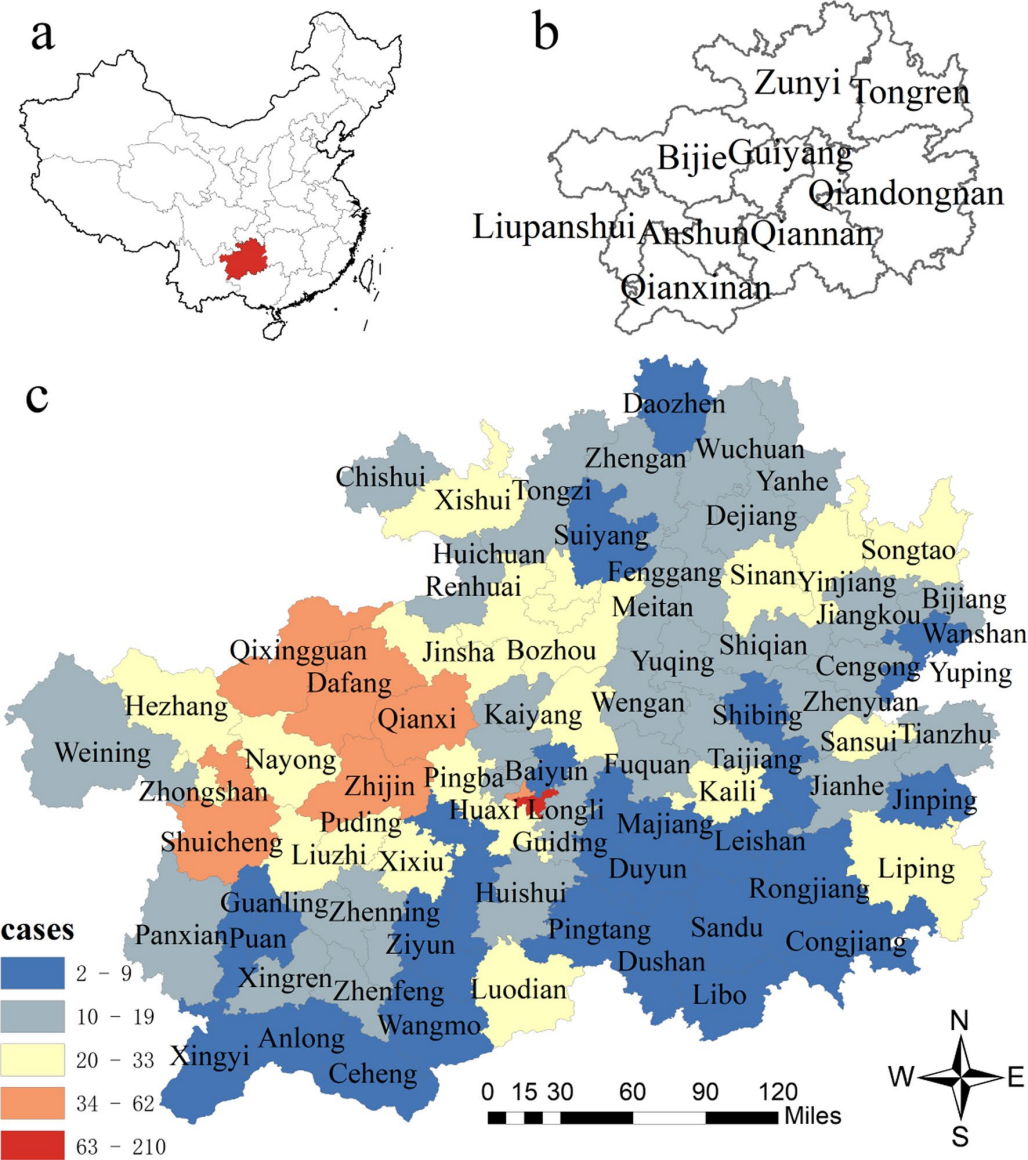
The geographical distribution of total MDR-TB cases in Guizhou from 2014 to 2020. **a** The geographical location of Guizhou in China; **b** The geographical location of 9 prefectures in Guizhou; **c** The geographical location of 88 counties in 9 prefectures, and distribution of total MDR-TB cases over seven years in each county

The number of MDR-TB cases in Guizhou based on prefecture level from 2014 to 2020 are shown in Table 5. Most cases were in Guiyang (400, 24%), Bijie (332, 20%) and Zunyi (245, 15%), with smallest shares in Qianxinan (57, 3%). Four prefectures had the higher average number of cases per county: Bijie (42), Guiyang (40), Liupanshui (27) and Zunyi (18). Figure 5 details that MDR-TB cases at county level have regional aggregation. The redder the colour is, the higher the number of cases is, and vice versa. There was high clustering in 7 counties, covering 2 counties of Guiyang (Nanming and Yunyan), 4 counties of Bijie (Qianxi, Dafang, Qixingguan, Zhijin) and 1 county of Liupanshui (Shuicheng). The descriptive analysis gave a rough impression that 4 prefectures (Bijie, Guiyang, Liupanshui, Zunyi) had higher MDR-TB burden than other prefectures. Clustering distribution is further tested by spatial autocorrelation analysis.

**Table 5:** The geographical distribution of MDR-TB cases in Guizhou Province, 2014–2020

Prefecture name	No. of cases								No. of county	No. of cases per county	County name
	2014	2015	2016	2017	2018	2019	2020	Total			
Bijie	20	33	23	53	44	53	106	332	8	42	Dafang Hezhang Jinsha Nayong Qixingguan Qianxi Weining Zhijin
Guiyang	56	75	110	43	33	36	47	400	10	40	Baiyun Guanshanhu Huaxi Kaiyang Nanming Qingzhen Wudang Xifeng Xiuwen Yunyan
Liupanshui	6	4	5	14	13	19	46	107	4	27	Liuzhi Panxian Shuicheng Zhongshan
Zunyi	25	18	24	50	58	40	30	245	14	18	Bozhou Chishui Daozhen Fenggang Honghuagang Huichuan Meitan Renhuai Suiyang Tongzi Wuchuan Xishui Yuqing Zhengan
Tongren	12	13	7	17	33	25	52	159	10	16	Bijiang Dejiang Jiangkou Shiqian Sinan Songtao Wanshan Yanhe Yinjiang Yuping
Anshun	9	1	1	17	5	15	32	80	6	13	Guanling Pingba Puding Xixiu Zhenning Ziyun
Qiandong nan	4	10	5	14	32	38	72	175	16	11	Cengong Congjiang Danzhai Huangping Jianhe Jinping Kaili Leishan Liping Majiang Rongjiang Sansui Shibing Taijiang Tianzhu Zhenyuan
Qiannan	9	10	8	10	15	35	24	111	12	9	Duyun Dushan Guiding Huishui Libo Longli Luodian Pingtang Sandu Wengan Changshun Fuquan
Qianxinan	4	1	3	7	7	8	27	57	8	7	Anlong Ceheng Puan Qinglong Wangmo Xingren Xingyi Zhenfeng
Total	145	165	186	225	240	269	436	1666	88	19

#### Global spatial autocorrelation

The global spatial autocorrelation analysis for MDR-TB suggested that the significant clustering distribution has been detected since 2014 at the county level in Guizhou, and the annual global Moran’s I reached the significance level of 0.05 (Table 6).

**Table 6:** Global spatial autocorrelation analysis

Year	Moran’s I	E(I)	S	Z-Value	p-Value
2014	0.297	− 0.011	0.004	4.799	0.000
2015	0.042	− 0.011	0.001	2.139	0.032
2016	0.019	− 0.011	0.000	2.002	0.045
2017	0.253	− 0.011	0.004	4.233	0.000
2018	0.218	− 0.011	0.004	3.485	0.000
2019	0.176	− 0.011	0.004	2.817	0.005
2020	0.341	− 0.011	0.004	5.483	0.000

#### Local spatial autocorrelation

**Fig. 6:**
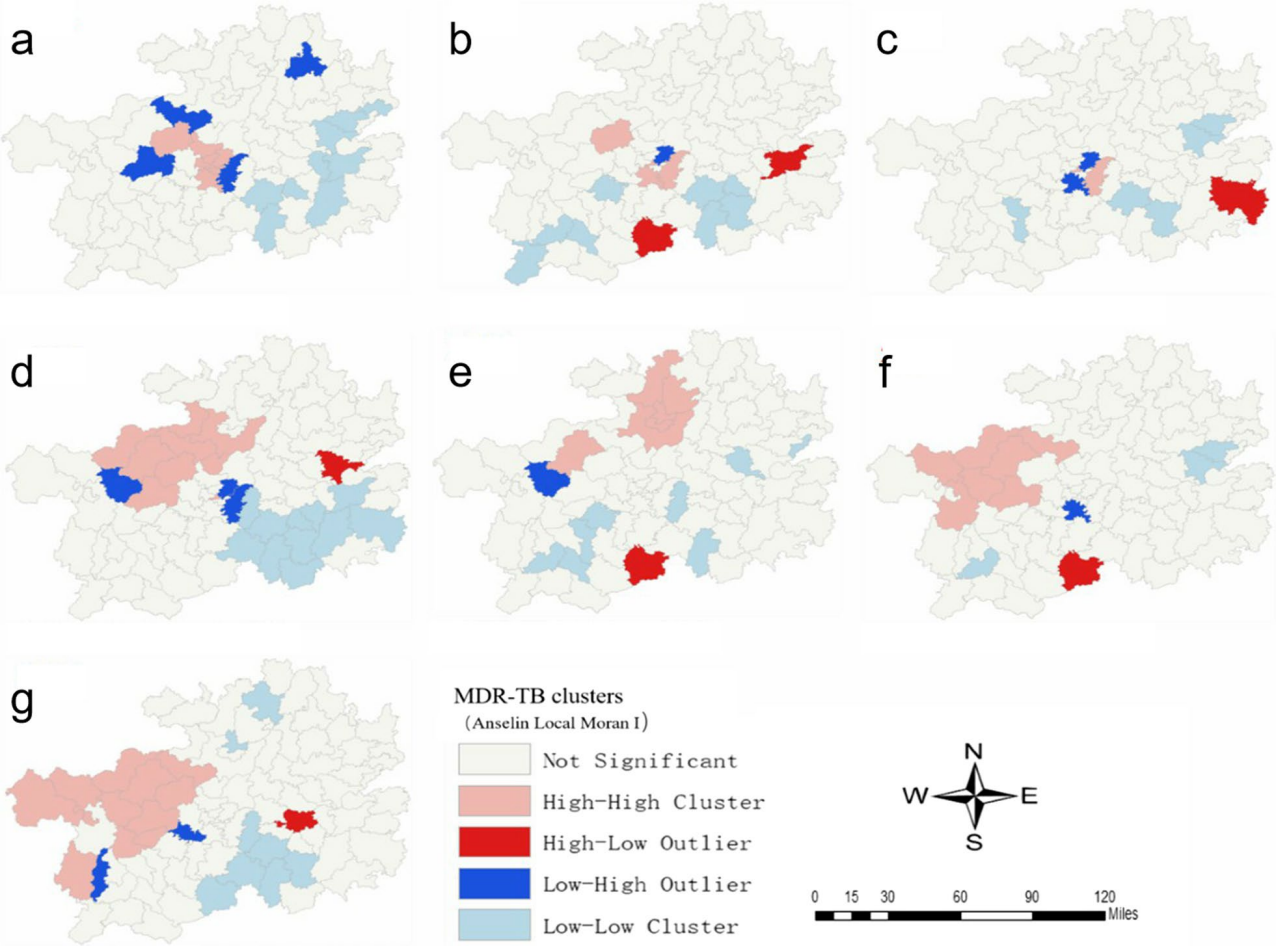
Maps of local spatial autocorrelation cluster of MDR-TB for each county in Guizhou province from 2014 to 2020 by ArcMap software. Only those counties whose local Moran’s I have reached the significance level of 0.05 will be present on the map: **a** MDR-TB clusters in 2014; **b** MDR-TB clusters in 2015; **c** MDR-TB clusters in 2016; **d** MDR-TB clusters in 2017; **e** MDR-TB clusters in 2018; **f** MDR-TB clusters in 2019; **g** MDR-TB clusters in 2020

Analysis from local spatial auto-correlation reveals that the detected MDR-TB HH clusters stably located in the northwest of Guizhou over the reporting years, mainly aggregating in Bijie, Guiyang, Liupanshui and Zunyi (Fig. 6). The results are basically consistent with those from the geographically descriptive analysis above. Meanwhile, Longli county in Qiannan prefecture was found as a HH cluster in 2015 and 2016, respectively. The results also showed that LL clusters consistently located in five prefectures in the southeast part, including Anshun, Qianxinan, Qiandongnan, Qiannan and Tongren. Yearly, LH clusters were scattered in 3 counties of Bijie (2014 Jinsha Zhijin, 2017 and 2018 Nayong), 2 counties of Guiyang (2015–2017 Wudang, 2016 Huaxi), Longli of Qiannan (2014 and 2017) and Dejiang of Tongren (2014). HL clusters appeared 3 counties of Qiandongnan (2015 Jianhe, 2016 Liping, 2017 Zhenyuan) and Luodian of Qiannan (2015 and 2018).

#### Spatial–temporal cluster analysis

**Fig. 7:**
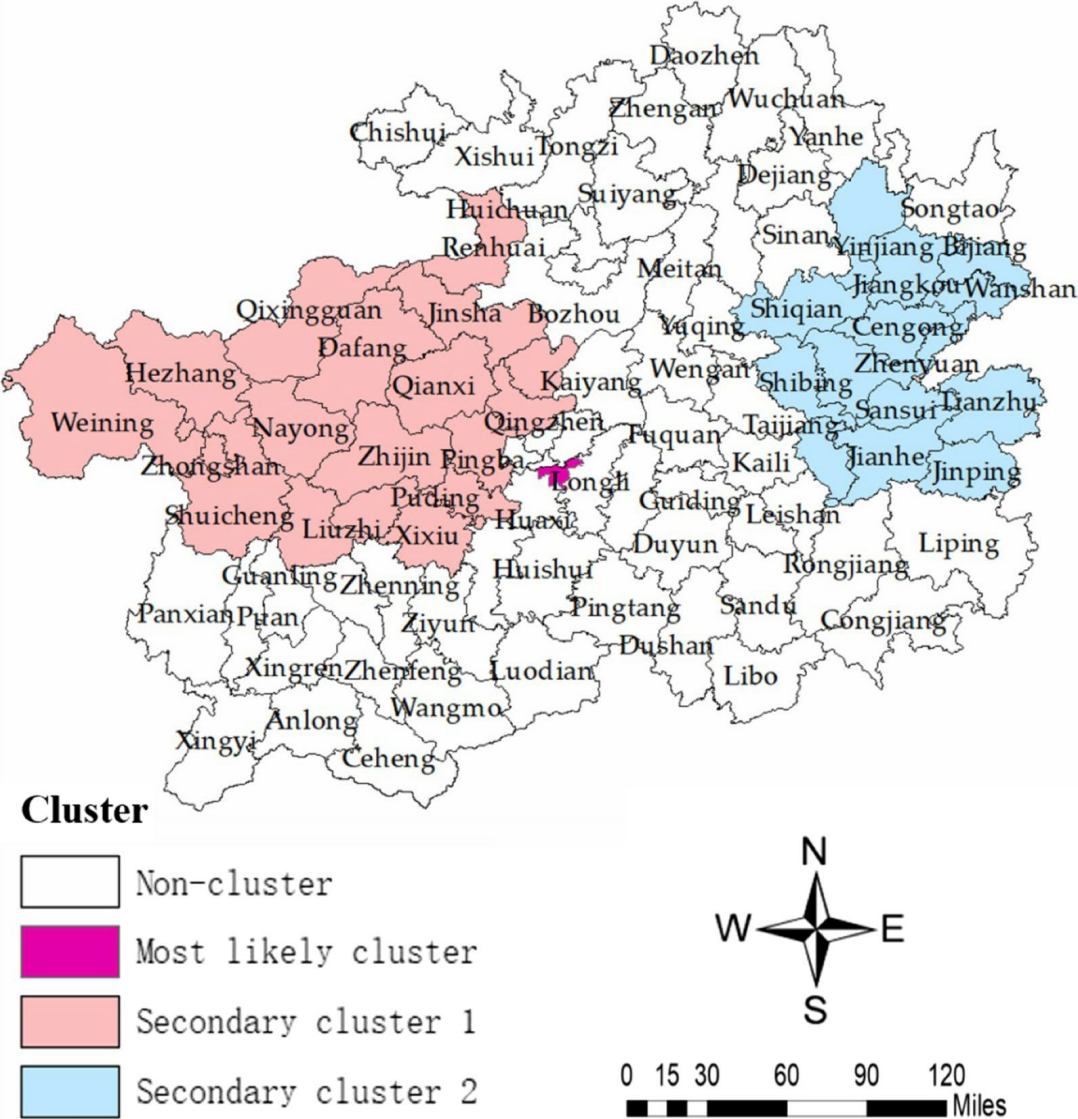
The detected spatial–temporal cluster map of MDR-TB cases in Guizhou (2014–2020)

Table 7 summarize the results of the retrospective space–time scan statistics of MDR-TB cases in different time frame and regions. One most likely cluster and two secondary clusters were shown in Fig. 7.

**Table 7:** Spatial − temporal scan of MDR-TB in Guizhou from 2014 to 2020

Time frame	Cluster type	No. and name of prefectures and counties	Center/radius (km)	Observed case	Expected case	LLR	RR	P-value
2015/1/1 to 2016/12/31	Most likely	Guiyang: Nanming	(26.57 N, 106.72 E) / 0 km	154	10.83	272.06	15.57	< 0.001
2020/1/1 to 2020/12/31	1st secondary	1. Bijie: Qixingguan, Dafang, Nayong, Hezhang, Qianxi, Zhijin, Jinsha, Weining 2. Guiyang: Xiuwen, Qingzhen, Xifeng 3. Liu panshui: Shuicheng, Zhongshan, Liuzhi 4. Zunyi: Renhuai 5. Anshun: Puding, Xixiu, Pingba	(27.30 N, 105.31 E) / 143.79 km	190	92.92	41.89	2.18	< 0.001
2020/1/1 to 2020/12/31	2st secondary	1. Tongren: Yuping, Jiangkou, Wanshan, Bijiang, Shiqian, Yinjiang 2. Qiandongnan: Cengong, Sansui, Tianzhu, Zhenyuan, Jinping, Jianhe, Shibing, Taijiang	(27.25 N, 108.92 E) / 97.11 km	71	23.04	32.65	3.17	< 0.001

*LLR* logarithmic likelihood ratio, *RR* risk ratio

The most likely cluster was detected from January 2015 to December 2016 with 154 reported cases (LLR = 272.06), covering 1 county (Nanming) in Guiyang prefecture. Compared with neighboring counties, Nanming had a 15.57 (RR = 15.57, P < 0.001) times higher cases in this cluster. Two statistically significant secondary clusters were detected with high cases of MDR-TB during 2020. The first secondary clusters with 190 reported cases (LLR = 41.89), distributed in 18 counties of five prefectures, including Bijie (Qixingguan, Dafang, Nayong, Hezhang, Qianxi, Zhijin, Jinsha, Weining), Guiyang (Xiuwen, Qingzhen, Xifeng), Liu panshui (Shuicheng, Zhongshan, Liuzhi), Zunyi (Renhuai) and Anshun (Puding, Xixiu, Pingba). These identified counties in this cluster had a 2.18 (RR = 2.18, P < 0.001) times higher cases than those outside of this cluster. The other secondary clusters with 71 reported cases (LLR = 32.65) covered 14 counties of two prefectures, which were Tongren (Yuping, Jiangkou, Wanshan, Bijiang, Shiqian, Yinjiang) and Qiandongnan (Cengong, Sansui, Tianzhu, Zhenyuan, Jinping, Jianhe, Shibing, Taijiang). These counties in this cluster had a 3.17 (RR = 3.17, P < 0.001) times higher cases than those outside of this cluster.

## Discussion

In this study, we first descriptively analyzed the general characteristics of MDR-TB in Guizhou from 2014 to 2020; then explored the temporal patterns and trend of these cases by SARIMA model; finally, clarified the spatial characteristics and spatial–temporal clustering at the county level by spatial−temporal analysis.

The general characteristics of cases in our study shows that MDR-TB mainly affects young to middle-age adult men without fixed salary. Similar results have been reported in WHO TB report [1] and a study conducted in Hunan of China [25]. One of serious consequences for this group after being diagnosed MDR-TB was losing job and productivity. Income loss and high direct medical costs for MDR-TB treatment pushed them into a catastrophic poverty-disease loop [36]. As a result, poor treatment adherence due to financial hardship promote patients further drug resistance, high mortality and transmission of this disease [36]. Another finding in our study provides the evidence that majority of patients were acquired MDR-TB, which was defined as a newly diagnosed MDR-TB patient received anti-TB therapy for at least 1 month before this diagnosis [37]. This is consistent with findings from Chinese studies [25, 37]. High acquired MDR-TB might be due to poor treatment adherence, inappropriate drug prescribing or pharmacokinetic variability [37, 38]. These findings in our study suggest that comprehensive strategies, such as financial and social protection, prescription supervision, should be taken to improve treatment adherence and the diagnosis measures on MDR-TB treatment.

The trend of MDR-TB cases increased dynamically during the years under our study, especially in 2020. The reason for the gradual increase in case notification was the gradual establishment and improvement of MDR-TB health service system and the efforts for case finding since 2014 in Guizhou [5, 39]. Meanwhile, a significant rise in notification in 2020 can be explained by two possible reasons. One reason was that the expanded implementation of active case finding starting from 2019 resulted in the current MDR-TB cases upward in 2020 [9]. The other reason was that the restrictions during COVID-19 pandemic have created barriers in the provision of healthcare and the treatment delay of patients [11, 40]. After lifting all restrictions in May in Guizhou [41], patients densely went to hospitals in June and led to a peak admission. While, the cases’ distribution with a peak in August in 2020 was similar to that in previous years. The 7 years of surveillance data in Guizhou exhibited a clearly seasonality and periodicity, which the trough usually occurred in February and most cases concentrated in autumn with a small decreasing from October. Studies conducted in China demonstrated that the PTB incidence reached a minimum in February [12, 14, 42]. This result concurs with our finding. The Spring Festival holidays, the most important traditional festival in China, may be a special reason for the significant decline of TB notification [12, 14]. In our study, the peak months of notification MDR-TB were later than other areas in China, such as Zhejiang [22], Guangxi [23] and Hunan [25]. These studies showed that the peak of PTB incidence ranged from March to September. The variation of PTB incidence might be influenced by many factors, such as number of health personnel, socio-economic, demographic and meteorological factors. [43–46]

Based on the rigorous spatial−temporal analysis in our study, the MDR-TB high risk areas mainly covered four prefectures located in the northwest of Guizhou. Among them, Bijie prefecture has the highest MDR-TB burden, largest population and lowest GDP per capita in Guizhou [6]. The backward economy, shortage of health resources and vast population may associate with a high MDR-TB infection in this region [24, 47]. Guiyang is the capital city and Zunyi is the second largest city of Guizhou. Two designated hospitals for MDR-TB treatment were first established in the two cities in 2012 [48, 49]. Thus, the following facts may result in high MDR-TB burden in the two prefectures: the central and urban areas, better MDR-TB diagnosis and treatment services, relatively convenient transportation facilities, neighbouring Bijie, having high population and immigration density [47, 49, 50]. While, with the gradual establishment of designated hospitals for MDR-TB in other prefectures, the number of patients reported in Guiyang has decreased since 2017. Liu panshui neighbour with Bijie and Guiyang. Relatively low economic level, geographical location and internal migration [50, 51] might, to some extent, contribute to high MDR-TB clustering in this prefecture. The number of MDR-TB cases in Anshun, Tongren and Qiandongnan in 2020 increased higher than previous years. The possible reasons may be similar to those explained above for the whole province. But more reasons are worth further exploration. The spatial−temporal distribution in our study provides the evidence that the prevalence of MDR-TB may be associated with geography.

Our study has limitations. First, the common problem of low enrollment for MDR-TB [1] may cause epidemic underestimation of this disease in Guizhou. Second, the data from surveillance system only contain limited variables. Thus, in this study, we could not assess those potential variables associated with clustering, such as elevation/topography, socio-economic [44] and meteorological [43, 45, 46] factors. The limitations will spur the future studies to improve the enrollment of MDR-TB, continuously monitor the trend of MDR-TB, comprehensively collect relevant influencing factors.

## Conclusion

This is the first study to analyze the temporal and spatial characteristics of MDR-TB in Guizhou. The results support our hypothesis that the reported MDR-TB cases showed an upward trend from 2014 to 2020. The expanded efforts of case findings contributed to an obvious and upward trend in 2020. COVID-19 pandemic restrictions may delay patients’ treatment which continued till the restrictions were lifted in May 2020. Hence, a peak admission was observed in June. The seasonal trends were detected with most cases during the autumn and the trough in February. The spatial–temporal heterogeneity revealed that MDR-TB cases stably aggregated in four prefectures (Bijie, Guiyang, Liupanshui and Zunyi) in the northwest over years. Three prefectures (Anshun, Tongren and Qiandongnan) only appeared cases cluster in 2020. The findings suggest that the epidemic characteristics among MDR-TB must be monitored continually and control efforts should target at high-risk periods and areas by prioritizing resources allocation to increase cases detection capacity and better access to treatment.

## Data Availability

The datasets generated and analysed during the current study are not publicly available due to the fact that it contains personal information, but are available from the corresponding author on reasonable request.

## Abbreviations

MDR-TB: Multidrug-resistant tuberculosis
PTB: Pulmonary tuberculosis
MTB: Mycobacterium tuberculosis
DST: Drug susceptibility testing
SARIMA: Seasonal autoregressive integrated moving average
AR: Auto-regressive
MA: Moving average
ACF: Autocorrelation function
PACF: Partial autocorrelation function
AIC: Akaike information criterion
BIC: Bayesian information criterion
HH: High–high
LL: Low–low
HL: High–low
LH: Low–high
LLR: Logarithmic likelihood ratio
Anti-TB: Anti-tuberculosis
RMSE: Root mean square derror
MAPE: Mean absolute percent error
LCL: Lower confidence limit
UCL: Upper confidence limit
